# Pupal melanization is associated with higher fitness in *Spodoptera exigua*

**DOI:** 10.1038/srep10875

**Published:** 2015-06-03

**Authors:** Sisi Liu, Mo Wang, Xianchun Li

**Affiliations:** 1 Department of Pesticide Science, College of Plant Science & Technology, Huazhong Agricultural University, Wuhan 430070, China; 2 Department of Entomology and BIO5 Institute, University of Arizona, Tucson, Arizona; 3 State Key Laboratory for Biology of Plant Diseases and Insect Pests, Institute of Plant Protection, Chinese Academy of Agricultural Sciences, Beijing 100193, China; 4 Hubei Insect Resources Utilization and Sustainable Pest Management Key Laboratory, Institute of Insect Resources, Huazhong Agricultural University, Wuhan 430070, China

## Abstract

Melanism has long been thought to be a habitat adaptation with a fitness cost. Here we reported a homozygous melanic strain (SEM) of *Spodoptera exigua* (Hübner) (Insecta: Lepidoptera: Noctuidae) established with black pupae spontaneously occurring within a typical laboratory population (SEW). The melanization is expressed globally, and only in the pupal stage. After pupation, the melanic SEM pupae gradually accumulate melanin to become completely black within 6 hours, whereas the wild-type SEW pupae gradually turn yellow-brown. The melanic SEM strain exhibits faster development in all life stages, heavier pupa weight, more mating time, higher fecundity, and accordingly, higher net reproductive rate and population trend index. While no reproductive isolation was observed between the SEM and SEW strains, the mating times per female of the reciprocal crosses and the SEM intracrosses were significantly higher than those of the SEW intracrosses. This represents a rare case of melanization that has fitness gains, rather than costs. Analysis of the life-history traits of this case and 14 previously reported cases of insect melanism indicate that none of melanization origin, stage, space and variation type determining whether melanism will cause fitness gain or cost.

Melanism is one of the most conspicuous external morphological variations among insect species^1^,^2^,^3^, populations of the same species^4^,^5^,^6^,^7^,^8^, and even individuals within a population^9^. It often occurs spontaneously at low frequencies, and can gradually reach high frequencies or fixation in response to natural selection for field populations^10^,^11^,^12^ or artificial selection for laboratory populations^13^,^14^. While holometabolous insects have four developmental stages, melanization is expressed only at one or two particular stages, including larval^4^,^7^,^15^,^16^, pupal^17^, adult^18^,^19^,^20^,^21^,^22^, larval and adult^8^,^17^, larval and pupal^23^, or pupal and adult stages^13^. In terms of spatial pattern, melanization is expressed either locally in a particular region of the body, commonly seen in Diptera^24^,^25^ and Coleoptera^26^,^27^, or globally in the whole body, more often in Lepidoptera.

In spite of the diversified origins/selection forces and spatiotemporal expression profiles, melanization has long been thought to be an environmental adaptation with a fitness cost in the absence of selections^20^,^28^,^29^,^30^. This fitness trade-off hypothesis is based on the premises that tyrosine, the precursor of melanin biosynthesis, can only be obtained from ingested food, and production of melanin requires substantial nitrogen or protein investment^28^,^31^, no matter how melanization reaches high frequencies or fixation and when/where it is expressed. Review of the previous papers that compared the life-history traits of the melanic vs. wild type morphs of eight insect species, however, showed that three out of the six melanic insects had a fitness gain, rather than fitness cost^30^. These include faster development and higher fecundity in *Mythimna separate*^21^, higher fecundity in *Malacosoma disstria*^18^, and faster development in *Biston betularia*^32^. Interestingly, two of the three melanic strains arose in wild populations through natural selection. By contrast, all the remaining three melanic insect strains were of laboratory origin through artificial selection and had fitness costs^30^. These include lower survival, lower fecundity, smaller body weight and slower development in *Manduca sexta*^4^, lower fecundity, smaller body weight and slower development in *Helicoverpa armigera*^13^, and lower fecundity in *Bicyclus anynana*^7^.

It appears whether a melanic strain originates through natural or laboratory selection is a key factor determining if melanism is associated with fitness gain or cost. To test if this observation represents a general trend, we established a homozygous pupal melanic strain with black pupae spontaneously occurring within a wild-type laboratory population of the beet armyworm, *Spodoptera exigua*. We observed and documented the time course of pigmentation in the pupal stage of both the wild type and melanic strains with a digital camera. We also compared the life history traits such as developmental speed, body weight, mating success rate, and fecundity of the two strains under the same laboratory conditions. The data obtained indicate that melanization resulted from shutting off of the brown pigment synthesis and early turning on of the melanin synthesis in the melanic strain. Our data also suggest that origin of melanism is not a key factor determining if melanism is associated with fitness gain or cost.

## Results

### Morphological photography

**Figure 1:**
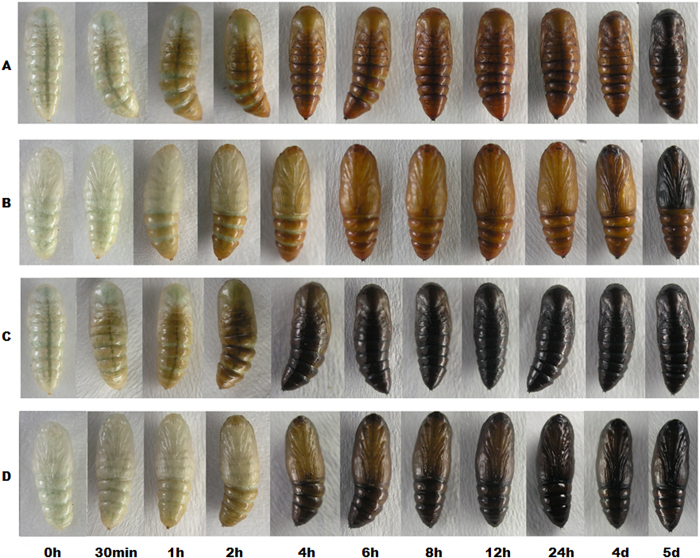
The time course of pigmentation in the wild type (SEW) and melanic (SEM) pupae of *Spodoptera exigua*. Photos were taken with the same individual of each strain across all of the time points. (**A**) SEW dorsal view. (**B**) SEW ventral view. (**C**) SEM dorsal view. (**D**) SEM ventral view.

Morphological observations show no color differences at egg, larval or adult stages. Melanization is globally expressed only at the pupal stage of the SEM strain (Fig. 1). Newly molted pupae (0 h) of both the melanic and wild type strain are light green in the head and milk-white in thorax and wing bud. Color changes first appear on the dorsal and ventral sides of both strains at 30 min and 1 h after pupation, respectively, with SEM pupae gradually blackening while SEW pupae gradually turning brown. By 4 h after pupation, the abdomens and wing buds of SEM and SEW pupae can be readily identified as black or brown, respectively. Pupae of SEM and SEW strains become completely dark and brown, respectively, at 12 h after pupation (Fig. 1), and remain unchanged in color for about 4 days. After that, SEW pupae gradually darken until eclosion, whereas SEM pupae exhibit no noticeable color changes (Fig. 1). Apparently, the time course of pupal pigmentation is the same in the two strains, but different pigments—black melanin and brown pigments—are deposited in SEM and SEW pupae, respectively.

### Life tables and life-history traits

Two-way ANOVA revealed that there were significant differences between the two strains in pupa weight, pupa duration and adult duration (Table 1). For each strain, there were significant differences between sexes in pupa weight and pupa duration. There were no significant strain-sex interactions in pupa weight, pupa duration and adult duration (Table 1).

Two-sample *t*-tests on all life-history traits indicated that larva duration and pupa duration of the SEM strain were significantly shorter than those of the SEW strain (Table 2). Compared with the SEW strain, the SEM strain had significantly heavier pupa weight in both sexes. The SEM strain also had significantly higher fecundity. However, no significant differences in egg duration, hatch rate, female duration and male duration were observed between the two strains (Table 2).

Chi-square tests found no significant differences in the mortalities during pre-pupation and eclosion (Table 3). But the mortalities from the first to third instar and from the fourth to fifth instar were significantly higher in the SEM strain than in the SEW strain. Because of higher larval mortality in the SEM strain, its total mortality was higher (65.28%) than that of the SEW strain (54.26%). Such a higher total mortality was overcompensated by SEM’s greater mating rate and mating times per female (Table 4) as well as its 2.48-fold greater fecundity (Table 3). As a result, the net reproductive rate (R_0_), relative fitness, and population trend index (*I*) of the SEM strain were 1.97-, 1.66- and 1.66- fold greater than those of the SEW strain, respectively (Table 3).

### Mating preference between the wild-type and melanic strains

The successful mating rate was higher in the two inter-strain crosses (♀SEW × ♂SEM and ♀SEM × ♂SEW) than in the two intra-strain crosses (♀SEM × ♂SEM and ♀SEW × ♂SEW), but the observed differences were not significant among the four types of crosses (Table 4). Similarly, the mating times per female were higher in the two inter-strain crosses than in the two intra-strain crosses. One-way ANOVA found significantly fewer mating times per female in ♀SEW × ♂SEW than in the other three types of crosses (Table 4). These indicated that inter-strain mating is preferred and no reproduction isolation exists between the SEW and SEM strains.

### Factors affecting fitness cost or gain in melanic insects

**Figure 2:**
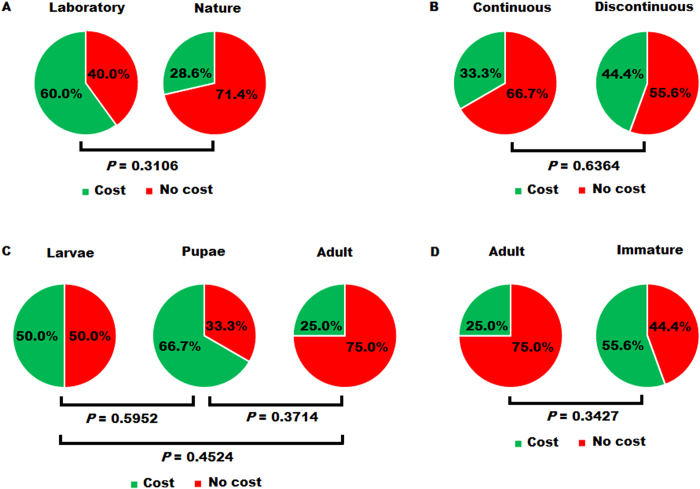
Fitness changes in melanic insects are independent of origin (A), variation type (B), and temporal expression pattern (C–D) of melanism. **A**. Laboratory (left, n = 5 cases) vs. nature (right, n = 7 cases) origin; **B**. Continuous (left, n = 3) vs. discontinuous (right, n = 9 cases); **C**. larvae (left, n = 6 cases) vs. pupae (middle, n = 3 cases) vs. adult (right, n = 4 cases); **D**. Adult (left, n = 4) vs. immature (right, n = 9). Fisher’s exact tests are performed to test if the left and right pies of each composite figure are significantly different from each other (See Table 5).

We further reviewed this study and 14 other cases for which fitness changes associated with melanism are published in peer-reviewed journals for 14 species that have a melanic strain or population (Table 5). Fitness variations were calculated according to the experimental data from 13 studies including this study. In five of the 12 species, the fitness of the melanic strain is significantly reduced relative to the wild type counterpart, but the fitness of the other seven species is significantly greater in the melanic strain than in the wild type strain (Table 5). Fitness cost is associated with melanism in three of five melanic species that are of laboratory origin, and in two of seven melanic species that are originated from nature (Fisher’s exact test, one-tailed *P* = 0.3106, Fig. 2A; Table 5). Four of nine melanic species with a discontinuous melanic vary type have a fitness cost, compared with one of three melanic species with a continuous melanic vary type (Fisher’s exact test, one-tailed *P* = 0.6364, Fig. 2B; Table 5). Fisher’s exact tests also indicate that fitness cost in melanic strains is independent of the expression stage (larvae vs. pupae, one-tailed *P* = 0.5952, pupae vs. adult, one-tailed *P* = 0.3714, larvae vs. adult, one-tailed *P* = 0.4524, Fig. 2C; and immature vs. adult, one-tailed *P* = 0.3427, Fig. 2D) of melanization.

## Discussion

While melanism is one of the most common conspicuous variations in insects, each melanic insect represents a unique case in terms of its origin, variation type, spatiotemporal expression pattern, and impacts on fitness. The pupal melanic mutant strain of *S. exigua* (SEM) we reported here was established by laboratory selection and, just like other laboratory-originated melanic Lepidoptera^4^,^7^,^13^,^21^, has a discontinuous variation type. Parallel observations of pigmentation across life stages in both the melanic (SEM) and wild type (SEW) strains that share a similar genetic background show that melanization is globally expressed only in the pupal stage. This is the second case of pupal melanism reported so far, and the first case of pupal melanism being *Inachis (Aglais) io,* which occurred from natural selection and had a continuous variation type^17^ (Table 5).

Parallel observations of the pigmentation time course in the pupal stages of both strains suggest that melanin is the major pigment synthesized/deposited in the SEM pupae (Fig. 1). By contrast, both brown and melanin pigments are synthesized/deposited in the SEW pupae, with brown pigments being synthesized/deposited at about the same pace as melanin in the SEM pupae within 24 h of pupation, and melanin being gradually synthesized/deposited approximately 4 days later. This cross-strain difference in pigmentation time course suggests that the biosynthetic steps leading to melanin and brown pigments are turned on and blocked/shut off, respectively, at the early puapl stage in the SEM strain. Based on current understanding of the biosynthetic pathway of insect pigments^33^,^34^, the candidate gene underlying melanization in the SEM strain is probably a melanin-promoting gene, such as tyrosine hydroxylase (TH) and *yellow* genes, or a transcription factor that enhances the stage-specific expression of this yet-to-be characterized melanin-promoting gene.

Contrary to the trade-off hypothesis between melanism and fitness^20^,^28^,^29^,^30^, the SEM strain exhibited significant fitness gains in most of life history traits including faster development in larval, pupal and adult stages, heavier pupa weight for both sexes, more mating times per female and higher fecundity (Tables 2 and 4). The only trade-off life history trait associated with the uncharacterized melanic allele(s) was higher larval mortality (Table 3). But overall, SEM had a fitness gain, rather than fitness cost, because its higher mortality was overcompensated by other traits, particularly its greater mating times per female (Table 4) and greater fecundity (Table 3).

SEM’s gain in the overall fitness contradicts the naturally-occurring pupal melanism of *I. io,* which had a fitness cost^17^ (Table 5). This is also in contrast with the laboratory-occurring pupal/adult melanism of *Helicoverpa armigera*, which had the similar pupal melanization phenotype and time course as SEM, but was associated with fitness cost^13^ (Table 5). However, this is consistent with laboratory-originated adult melanism in *M. separate*^21^,^35^, naturally-occurring adult melanism in *Malacosoma disstria*^18^ and *Drosophila immigrans*^25^, and naturally-occurring larval melanism in *Spodoptera littoralis*^36^ and *Araschnia levana*^17^. After reviewing eight case studies, Roff & Fairbairn have recently proposed that melanism of natural origin is likely to have a fitness gain, whereas that of laboratory origin is likely to have a fitness cost^30^. Our analysis of 15 cases for which fitness changes associated with melanism are published in peer-reviewed journals (Table 5) does not support this proposition (Fig. 2A). Our Fisher’s exact tests further reveal that fitness cost in melanic insects is also independent of the expression stage and variation type of melanization (Fig. 2). Although the number of cases analyzed is relatively small, these tests suggest that fitness changes in melanic insects are probably case-specific and difficult to predict by the origin, variation type and spatiotemporal expression pattern of melanism.

Inter- and intra- crosses not only show that there is no reproductive isolation between the two strains, but also demonstrate that the SEM strain prefers to mate with the SEW strain as both the mating rate and the mating times per female were higher in the two intra-strain crosses than in the two inter-strain crosses (Table 4). Wing color and melanization are known to play a role in mate choice and species recognition in *Pieris* butterflies^37^,^38^,^39^ and *Colias* butterflies^40^. The question is how SEM adults differentiate themselves from SEW moths since melanization is not expressed in the adult stage. Characterization of the genetic alterations responsible for the melanism in the SEM strain may resolve this question and provide insight into SEM’s mate preference and higher fitness.

## Methods

### Experimental animals

The wild-type laboratory strain (named SEW) of *S. exigua* was established with a collection from cotton fields in Jingzhou, Hubei in 2003. At the sixth generation, 40 black pupae spontaneously occurred within the SEW strain. The adults that emerged from the 40 black pupae were crossed to establish the pupal melanic strain (named SEM). Both strains were reared under the conditions of 27 ± 1 °C, 75% humidity and photoperiod of 14:10 h (L:D) on artificial diets. The artificial diets are a solid mixture of 10% (M/V) soybean flour, 7.5% (M/V) barley flour, 3.13% (M/V) yeast powder, 0.63% (M/V) ascorbic acid, 0.22% (M/V) benzoic acid, 0.22% (M/V) sodium benzoate, 1.25% (V/V) acetic acid and 1.25% agar (M/V). The pupae of the wild type SEW strain are light brown in color, whereas the pupae of the melanic SEM strain are globally black.

### Observation and photography of the pupal pigmentation

To reveal the pigmentation differences between the SEW and SEM strains, we observed and documented the pigmentation time courses of the SEW and SEM pupae with a digital camera (Olympus Corp., Camedia C-5060, Japan) under white light. The pupae of both strains were observed and pictured immediately after pupation (zero time point), once per 15 min in the first two hours post pupation, once per 2 hours from 2 to 12 hours, once per 4 hours from 12 to 24 hours, and then once per day until eclosion.

### Construction of life tables and measurements of life-history traits

We started the life-table experiments of both the SEW and SEM strains by setting up 3 mating cages (i.e. n = 3 replicates) of 20 pairs of virgin adults each for each strain. When the females in each cage began to lay eggs on a piece of cheesecloth placed on the top of the cage, we collected the eggs daily by replacing the cheesecloth. About 200 fertilized eggs on the cheesecloth from each replicate cage were placed into a 9-cm petri dish. The eggs were checked daily until hatching to calculate the egg stage duration. The numbers of neonates hatched were recorded to calculate the hatching rate.

One hundred forty-four neonates that hatched on the same day from each of the 3 replicate petri dishes per strain were randomly picked and individually transferred with a soft paintbrush into clean glass tubes (Φ2 cm × 10 cm, 1 neonate per tube) containing artificial diets. We replaced old diets with fresh diets and recorded the survivorship and development stage of each individual daily until pupation. We sexed and weighed each pupa 3 days after pupation. Then, we put each pupa back into the original tube and recorded their survivorship and development until emergence.

For each strain, we set up 30 mating cages (n = 30) each containing a pair of virgin adults emerged from the above tubes on the same day. We checked the survivorship of the adults in each cage and counted the number of eggs laid daily until both the male and female died to calculate the lifespan of adults and fecundity per female. After each female died, we dissected the female and counted/recorded the number of spermatophores it had, which was equal to the mating times of each female.

### Inter- and Intra-strain crosses

In order to test whether there was reproductive isolation or mating preferences between the melanic SEM and wild type SEW strains, we conducted 2 types of intra-strain crosses (♀SEW × ♂SEW and ♀SEM × ♂SEM) and 2 types of inter-strain crosses (♀SEW × ♂SEM and ♀SEM × ♂SEW). For each type of crosses, we set up 16 mating cages (n = 16), each containing a single pair of virgin adults randomly taken from the corresponding strain(s). We collected eggs from each cage daily until the female died and checked to see if the collected eggs hatched into live neonates. We calculated the mating rate by dividing the number of cages producing live neonates by the total number of mating cages for each cross (i.e. n = 16). The dead females from the successful mating cages of each cross were dissected to determine how many times they mated by counting the number of spermatophores in their seminal receptacle. The mating times per female were then calculated by dividing the total number of spermatophores by the number of female having spermatophores.

### Statistical tests of differences in life history traits between the two strains

Cross-strain differences in pupa duration, pupa weight and adult duration were analyzed by two-way analysis of variance (ANOVA) with strain and sex as the main factors. Differences in other life history traits including pupa weight, pupa duration, adult duration, fecundity, egg hatch rate, egg duration and larval duration between the two strains were evaluated by two-sample *t*-tests with the Bonferroni correction. Differences in mating rate among the four inter- and intra- strain crosses were evaluated by Fisher’s exact test, and differences in mating times per female among the four inter- and intra- strain crosses were evaluated by one-way ANOVA followed by Tukey HSD multiple comparisons. Cross-strain differences in the mortality of each stage were evaluated by chi-square tests. All the above statistics were performed with SPSS version 16.0 (SPSS Inc., Chicago, IL, USA).

### Statistical tests of factors affecting fitness cost of melanism

We collected the fitness data of the melanic morph relative to the wild type morph from this study and 15 studies published as of 2014 (Table 5). The life-history traits included larva duration (L.D.), larva weight (L.W.), survival rate (S.R.), pupa weight (P.W.), adult size (A.S.), mating rate (M.R.), mating times (M.T.) and fecundity (F). The mean values of these traits were collected from the corresponding references. Fitness variations in duration of a given stage such as L.D. were calculated with the formula^41^,42^42^ of (L.D._non-melanic_- L.D._melanic_) / L.D._non-melanic_ × 100%, whereas fitness changes in all other life history traits were obtained by the formula^41^,^42^ of (L.W._melanic_− L.W._non-melanic_) / L.W._non-melanic_ × 100%. For each melanic case, if all the tested life-traits were significantly lower or higher in the melanic strain, we called a cost or gain in the overall fitness. For the cases where life-history traits varied in both up and down directions, we called a cost or gain in the overall fitness based on the fecundity change or the sum of changes in all the tested fitness parameters when the fecundity was not tested.

We divided the overall fitness changes of the 12 insect species into three classes: no change (i.e. equal overall fitness between the two morphs), fitness cost (significant reduction in the overall fitness of the melanic mroph), and fitness gain (significant increase in the overall fitness of the melanic morph). We used Fisher’s exact test (http://graphpad.com/quickcalcs/contingency1.cfm) to test the null hypothesis that the fitness of the melanic strain relative to the wild type counterpart was independent of the origin (natural vs. laboratory), expression stage (larvae vs. pupae, pupae vs. adult, larvae vs. adult, and immature vs. adult) or variation type (continuous vs. discontinuous) of melanization. In these analyses, fitness changes were divided into two classes: 1) causing no fitness cost (i.e. equal fitness + fitness gain) and 2) causing fitness cost.

## Additional Information

**How to cite this article**: Liu, S. *et al.* Pupal melanization is associated with higher fitness in *Spodoptera exigua*. *Sci. Rep.* **5**, 10875; doi: 10.1038/srep10875 (2015).

## Figures and Tables

**Table 1:** Two-way ANOVA on three life-history traits between the SEW and SEM strains of *S. exigua*.

**Life-history traits**	**Source of variation**	**d.f.**	**MS**	**F**	***P*-value**
Pupa weight	Sex	1	6666.395	32.067	0.000
	Strain	1	23800.372	114.486	0.000
	Sex × Strain	1	7.673	0.037	0.848
	Error	390	207.889		
Pupa duration	Sex	1	46.532	69.499	0.000
	Strain	1	9.108	13.604	0.000
	Sex × Strain	1	0.096	0.144	0.705
	Error	215	0.670		
Adult duration	Sex	1	0.012	0.002	0.962
	Strain	1	33.440	6.321	0.014
	Sex × Strain	1	2.012	0.380	0.539
	Error	80	5.290		

**Table 2:** Differences in life-history traits between the SEW and SEM strains of *S. exigua*.

**Life-history traits**	**Strains**		
	**SEW**	**SEM**	***P*-value**
Egg duration (day)	2.61 ± 0.07	2.40 ± 0.16	0.422
Hatch rate (%)	96.16 ± 1.85	97.76 ± 1.44	0.277
Larva duration (day)	17. 34 ± 1.10	16.49 ± 1.02**	<0.0001
Pupa duration (day) ♀	7. 27 ± 0.80	6.79 ± 0.89*	0.0018
Pupa duration (day) ♂	8. 38 ± 0.76	7. 76 ± 0.77**	<0.0001
Pupa weight (mg) ♀	123.60 ± 12.34	139.01 ± 18.38**	<0.0001
Pupa weight (mg) ♂	115.01 ± 11.91	130.99 ± 15.22**	<0.0001
Adult duration (day) ♀	7.86 ± 2.59	6.29 ± 2.31	0.0445
Adult duration (day) ♂	7.52 ± 2.27	6.57 ± 1.99	0.156
Egg number per female	256.70 ± 146.13	637.10 ± 234.34**	0.0002

^*^Significant difference at *P* < 0.05 / 10 = 0.005 (multiple two-sample *t*-tests, Bonferroni correction).

^**^Significant difference at *P* < 0.01 / 10 = 0.001 (multiple two-sample *t*-test, Bonferroni correction).

**Table 3:** The life tables of the SEW and SEM strains of *S. exigua*.

**Development period**	**Strains**		**x^2^**	***P*-value**
	**SEW**	**SEM**		
Initial number of neonates	384	433	-	-
Mortality from first to third instars	33%	42%	6.54	0.011
Number of dead 1st-3rd instars larvae	128	182	-	-
Number of 4th instars larvae	256	251	-	-
Mortality from fourth to fifth instars	8.59%	16.73%	7.612	0.006
Number of dead 4th-5th instars larvae	22	42	-	-
Number of pre-pupae	234	209	-	-
Mortality during pre-pupation	9.83%	5.26%	3.248	0.072
Number of dead pre-pupae	23	11	-	-
Number of pupae	211	198	-	-
Mortality during eclosion	2.84%	1.29%	0.838	0.360
Number of dead pupae	6	3	-	-
Number of adults	205	195	-	-
Number of females	116	86	-	-
Sex ratio (female:male)	1.3:1	1:1.26		
Average egg number per female^1^	257	637	-	-
Hatching rate (%)	96.2	97.8	-	-
Predicted number of neonates of next generation^2^	32338.7	53576.8	-	-
Net reproductive rate (R_0_)^3^	139.74	275.66	-	-
Population trend index (*I*)^4^	74.69	123.73	-	-
Relative fitness^5^	1	1.66	-	-

^1^Average egg number per female: average number of the fertilized eggs laid by female from single pair mating.

^2^Predicted number of neonates of next generation of SEW = Number of females × average egg number per female × hatching rate × initial number of SEM neonates/ initial number of SEW neonates; Predicted number of neonates of next generation of SEM = Number of females × average egg number per female × hatching rate × initial number of SEM neonates.

^3^net reproductive rate (R_0_) of SEW = Predicted number of neonates of next generation of SEW × female ratio/(number of female × initial number of SEM neonates/initial number of SEW neonates); net reproductive rate (R_0_) of SEM = Predicted number of neonates of next generation of SEM × female ratio/number of female.

^4^Population trend index (*I*) of SEW = Predicted number of neonates of next generation of SEW/initial number of neonates of SEW × initial number of SEM neonates/initial number of SEW neonates; Population trend index (*I*) of SEM = Predicted number of neonates of next generation of SEM/initial number of neonates of SEM.

^5^Relative fitness = *I*_SEM_/*I*_SEW._

**Table 4:** Differences in mating rate between the intra- and inter-strain crosses of the SEW and SEM strains of *S. exigua*.

**Mating conduction**	Mating rate (%)*	**Mating times per female**‡
♀SEW×♂SEM	93.80 a	2.40 ± 1.30 a
♀SEM×♂SEW	87.50 a	2.43 ± 1.22 a
♀SEM×♂SEM	84.81 a	1.97 ± 1.01 ab
♀SEW×♂SEW	74.68 a	1.44 ± 0.62 b

^*^Data with the same letters are not significant (Fisher’s exact test *P* < 0.05).

^‡^Mating times per female = the total number of mating times per group/the total number of females having spermatophores per group. Data with different letters are significant (Tukey HSD multiple comparison, *P* < 0.05).

**Table 5:** Summary of insect melanism variation and life-history changes.

**Species**	**Origin**	**Stage**	**Variation type**	**Space**	**Life-traits**	**Fitness(%)**	**Overall variation**
Lepidoptera							
*Manduca sexta*^4^	Laboratory	Last instar larvae	Discontinuous	Global	L.D.	−20.00	Cost
					P.W.	−32.02	
					F.	−51.58	
*Bicyclus anynana*^7^	Laboratory	Larvae	Discontinuous	Global	L.D.	3.03 N.S.	Cost
					P.W.	6.67 N.S.	
					M.R.	−25	
*Spodoptera exigua*	Laboratory	Pupae	Discontinuous	Global	L.D.	4.9	Gain
					P.W.	13.16	
					F.	148.19	
					M.R.	25.6 N.S.	
					M.T.	66.67	
					S.R.	−31.74	
*Helicoverpa armigera*^13^	Laboratory	Pupae/Adult	Discontinuous	Global	L.D.	−4.47	Cost
					P.W.	−4.42	
					F.	−45.05	
					M.R.	−6.70 N.S.	
					M.T.	−60.52	
					S.R.	−12.39 N.S.	
*Mythimna separata*^21^	Laboratory	Adult	Discontinuous	Global	L.D.	4.52	Gain
					F.	21.43	
					S.R.	7.51 N.S.	
*Galleria mellonella*^43^	Natural	Larvae	Discontinuous	Global	L.D.	2.65	Cost
					P.W.	−30.87	
					F.	−51.65	
*Spodoptera littoralis*^15^,^36^	Natural	Mid-6^th^ instar larvae	Continuous	Global	L.W.	9.38	Gain
					L.D.	6.58	
					P.W.	3.44	
					L.W†	−12.00	
*Papilio polyxenes*^16^	Natural	5^th^ instar larvae	Discontinuous	Partial	L.D	21.43	Gain
*Araschnia levana*^17^	Natural	5^th^ instar larvae	Continuous	Global	L.D.§	19.25	Gain
					S.R.	5.96	
					A.S.§	3.56	
*Inachis io*^17^	Natural	Pupae	Continuous	Global	L.D.§	−6.47	Cost
					A.S.§	−2.69	
*Odontoptera bidentata*^19^	Natural	Adult	Discontinuous	Global	Slower in adult stage		
*Malacosoma disstria*^18^,^22^	Natural	Adult	Discontinuous	Global	P.W.	5.39 N.S.	Gain
					F.	15.07	
					P.W‡	−10.34	
					A.S‡	−3.75	
*Biston betularia*^11^	Natural	Adult	Discontinuous	Global	Declined frequency		
Diptera							
*Drosophila polymorpha*^24^	Natural	Adult	Discontinuous	Partial	Higher desiccation resistance		
*Drosophila immigrans*^25^	Natural	Adult	Discontinuous	Partial	F	51.21	Gain

N.S.: No significance.

L.D.: larva duration; L.W.: larva weight; S.R.: survival rate; P.W.: pupa weight; A.S.: adult size; M.R.: mating rate; M.T.: mating times; F.: fecundity.

^†^This data was from the reference 15 and the rest data of this case were from reference 36.

^§^The mean values were calculated with the log-transformed data in the figure with the exponential function of e.

^‡^These data were from the reference 22 and the mean values of A.S. were calculated from the figure in the reference and ratio was reported in the reference.
